# A time-independent, variational method for studying the photodissociation of triatomic molecules

**DOI:** 10.1039/d4cp02771j

**Published:** 2024-10-18

**Authors:** Marco Pezzella, Georgi Mitev, Sergei N. Yurchenko, Jonathan Tennyson, Alexander O. Mitrushchenkov

**Affiliations:** a Department of Physics & Astronomy, University College London London WC1E 6BT UK j.tennyson@ucl.ac.uk; b MSME, Université Gustave Eiffel, CNRS UMR 8208, Univ Paris Est Creteil F-77474 Marne-la-Vallée France

## Abstract

The photodissociation of molecules is becoming an increasingly important factor to consider in the evolution of exoplanets' atmospheres orbiting around UV-rich stars, as it leads to the enrichment of atmospheric complexity. A new method is developed for computing the rotationally and vibrationally resolved photodissociation spectrum of triatomic molecules. The time-independent Schrödinger equation is solved using the variational nuclear motion program Everest; a new code Exocsmooth is employed to compute the cross-sections by applying Gaussian smoothing to a set of discrete transitions into the continuum. HCN is chosen as the test molecule, as it has been widely studied in the literature. Results are compared with the available experiments. Temperature dependence is explored for temperatures up to 2000 K.

## Abstract 2

The photodissociation of molecules is becoming an increasingly important factor to consider in the evolution of exoplanets' atmospheres orbiting around UV-rich stars, as it leads to the enrichment of atmospheric complexity.

## Graphical abstract

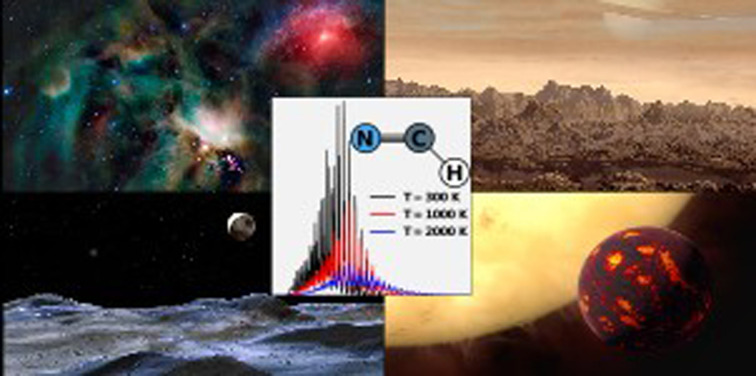

## 1 Introduction

Understanding the photochemistry of planetary atmospheres is becoming extremely important in this new revolutionary era of astronomy. The recent discovery of photochemically produced SO_2_ in WASP-39b is an example where photochemical processes, including photodissociation, play a crucial role in explaining the complexity of atmosphere composition and dynamics, as well as non-LTE effects.^1,2^ Exoplanets orbiting near M-dwarfs, small oxygen-rich stars with temperatures between 2300 and 3800 K which are the commonest stars in our galaxy, experience high UV fluxes of photons from their host stars.^3–6^ Due to the planets’ proximity to the star, they have temperatures on the order of magnitude of thousands Kelvin, generating vibrationally and rotationally excited molecules. Challenges arise in collecting photodissociation cross-sections at these high temperatures,^7^ due to the lack of experimental and computational data. Experiments struggle to reach the temperatures required to model the top of hot atmospheres, and calculations often assume a harmonic ground state wave function.^8^ As a reflection of these two factors, standard databases contain data for molecules at interstellar temperatures (close to 0 K).^9,10^

In recent years, cross-section calculations have begun to include rovibrational excitation of molecules as a function of temperature.^11^ The ExoMol database^12,13^ was designed to produce comprehensive line lists of hot bound–bound transitions for molecules present in exoplanets’ atmospheres and is expanding to include photodissociation data. Previous studies^14^ have demonstrated that the photodissociation of diatomic molecules can be simulated using the program Duo,^15^ which was originally developed to solve the bound–bound nuclear motion problem for diatomics allowing for different types of couplings and crossings between potential energy curves. Duo uses a grid-based variational solution of the Schrödinger equation based on the use of a sinc-DVR basis which has been shown to be highly accurate^16^ and which results in a discretised representation of the continuum. ExoCross^17^ is used to generate the raw cross-section for different grid sizes. The results obtained using different radial grids and smoothing the results with an appropriate Gaussian function generate spectra comparable with those of experiments. This methodology was successfully applied to produce photodissociation line lists for HCl and HF;^18^ these results were subsequently corroborated by Qin *et al.*^19^

The transition from two to three atoms adds complexity to the computations involved in generating photodissociation cross-sections, not least because the continuum now becomes multi-dimensional. Determining the best set of internal coordinates for the given potential energy surfaces and defining the transition dipole moments are non-trivial tasks that depend on the chemical species under examination. HCN was selected as the initial molecule to assess the generation of photodissociation cross-sections for triatomic molecules as at lower energies it has a clear set of photodissociation products: H + CN. HCN has an ubiquitous presence in space; it is detected in the interstellar medium,^20–24^ comets,^25–27^ planets,^28–30^ exoplanets^31^ and, finally, in the atmosphere of Titan,^32^ where it is supposed to be a main reservoir of carbon.^33,34^ The HCN/HNC ratio is very sensitive to stellar UV flux^35,36^ and can be used as a tracer for the heating of the nebular molecular gas by UV photons.^37–39^

The photochemistry of HCN has undergone extensive examination, encompassing both experimental and computational approaches. Experiments explored dissociation from the predissociative 2 ^1^A′ ← 1 ^1^A′ transitions at various wavelengths, successfully reconstructing the potential dissociation mechanism.^40–43^ Dissociation events from 1 ^1^A′′, 2 ^1^A′′, and 2 ^1^A′ states involve the cleavage of the H–CN bond through bending progression.^44^ Analysis of the electronic state distribution of the CN fragment, originating from the bending of the 3 A′ ← 1 A′ transition, indicates potential intersystem crossing and/or internal conversion between various excited states.^45^ This hypothesis has been corroborated by measurements from ^1^A′(*v*_CH_ = 3) to the 1 ^1^A′′ electronic states: the lack of rotational population suggests that the bond breaks when HCN is in the linear arrangement.^46^

The ground state potential energy surface (PES) has been computed by various research groups,^47–52^ whereas surfaces from the excited states are relatively scarce. The first study of excited states was published by Perić *et al.*^53^ in 1987. This work was followed by studies performed at the University of New Mexico between 2001 and 2003 on predissociation from the 1 ^1^A′′ (ref. 54–56) and 2 ^1^A′ (ref. 57) electronic states. Vibrational predissociation resonances in the latter state accelerate the dissociation due to efficient energy flow facilitated by an accidental coincidence between H–C and C–N vibrational normal modes. Nayak and collaborators calculated a variety of excited singlet states,^58^ followed by the work from Chenel *et al.*,^59^ which was used to explore the rates in the interstellar medium.^60^ The triplets states have been recently observed by Priyadarshini and coworkers.^61^

An alternative method to generate photodissociation cross-sections is through the use of the time-dependent Schrödinger equation. In this approach, the cross-section is proportional to the Fourier transform of the time-dependent autocorrelation function of the time evolution of the wave packet in an excited electronic state. Among all time-dependent methodologies, the multi-configurational time-dependent Hartree (MCTDH) approach, developed by Meyer *et al.*,^62^ stands out as one of the most versatile methods, as demonstrated by multiple improvements to the original code and its various implementations.^63,64^ The first photodissociation application appeared, for NOCl^65^ and NO_2_,^66^ two years after its introduction. These studies were quickly followed by investigations of the photodissociation of larger molecules, such as methyl iodide, which involved five nuclear degrees of freedom across three excited electronic states.^67,68^ The recent perspective from Han *et al.*^69^ discusses the most up-to-date applications of the method.

Although acknowledging the advantages of time-dependent methods for studying photodissociation, we opt for a time-independent approach for three reasons: (1) the ability to generate state-to-state photodissociation spectra, which are useful for modelling non-LTE effects, (2) the convenience of applying it to indirect photodissociation processes, as it is less time-intensive than the time-dependent approach^70^ and (3) it allows the use of a single, often complicated, model for all photon absorption processes,^71^ which includes the calculation of photoabsorption cross-sections across an extended wavelength range.^72,73^

In this work, we explore the ability to extend the approach used for diatomics to study continuum processes in larger systems. The DVR3D^74^ and Everest^75^ codes were chosen because they have been successfully used to describe bound–bound electronic transitions in previous studies, as demonstrated by Zak *et al.*^76^ and Owens *et al.*,^77^ respectively.

This paper is organised as follows: in the Methods section, a description and tests of our approach, the potential energy surface used, the choice of the basis, and the cross-section calculation are discussed. In the Results section, we compared our cross-section and the integrated intensities with previous experiments and calculations. Finally, the temperature dependence of photoabsorption is explored for three different temperatures.

## 2 Methods

### 2.1 Potential energy and transition dipole moment surfaces

**Fig. 1:**
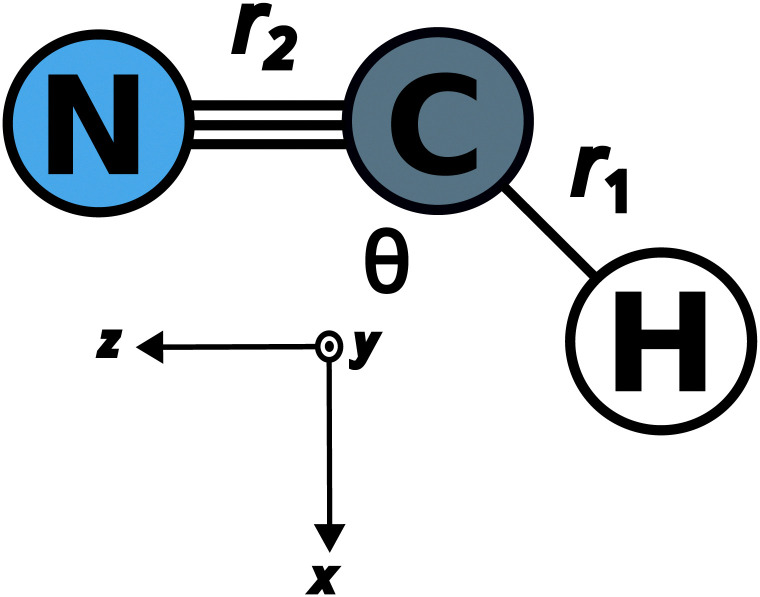
BLBA coordinates of HCN are used in this work. The reference axis, shown below the molecule, indicates the transition dipole moment orientation.

Potential energy surfaces for this work are taken from the literature. The ground state has been taken from the study by Makhnev *et al.*^52^ and chosen for its spectroscopic accuracy when compared to experiments. The PESs and transition dipole moment surfaces (TDMS) for the excited A′ states have been provided through private communication with Chenel *et al.* (16ChRoAg).^59^ The potential energy surfaces are represented using the bond-length-bond-angle (BLBA) representation with the following the convention: *r*_1_ = *r*_CH_, *r*_2_ = *r*_CN_ and *θ* = HĈN. The transition dipole moments are oriented such that the *z*-axis is aligned to *r*_CN_, the *x*-axis is perpendicular to the *z*-axis inside the molecular plane, and the *y*-axis component, which has a zero contribution, is perpendicular to the molecular plane. Fig. 1 shows the molecular coordinates and the reference axis of the transition dipole moment.

**Fig. 2:**
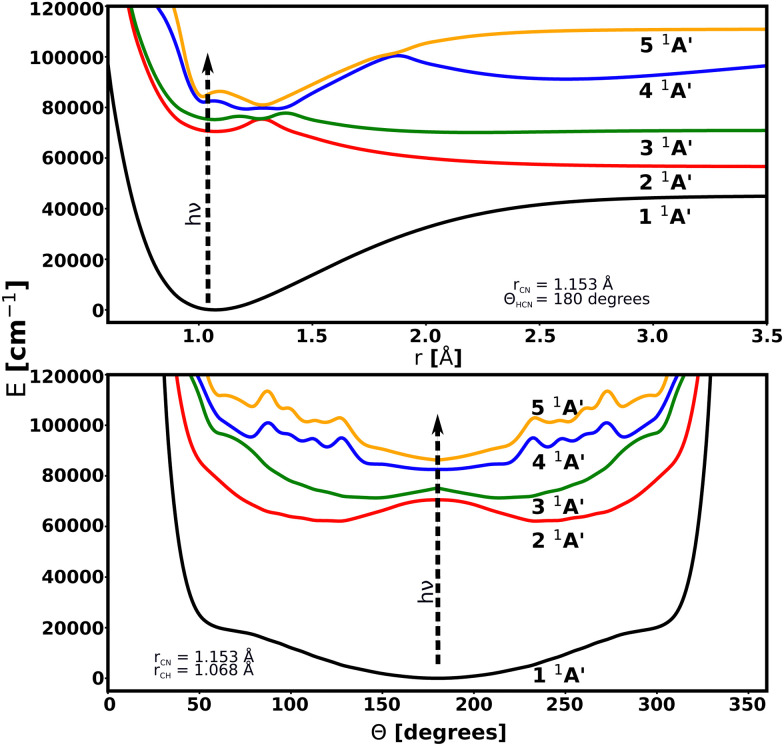
Cuts through the potential energy surfaces of the low-lying singlet electronic states of HCN considered in this work as a function of *r*_CH_ (up) and *Θ* (bottom).

Transitions from the 1 ^1^A′ ground electronic state to the 2 ^1^A′, 3 ^1^A′, 4 ^1^A′, and 5 ^1^A′ excited states are considered. 2 ^1^A′ and 3 ^1^A′ states are mainly dissociative states with a bound region near the linear structure due to an energy barrier along the *r*_CH_ coordinate for both surfaces. No transitions between excited states are included because of their large excitation energies. The PESs are illustrated in Fig. 2. Details of these PESs are shown in Table 1.

**Table 1:** Linear configuration mapping, excitation energies (*T*_0_) and vertical excitation energies (Δ*E*) for the HCN electronic states considered in this work. *T*_0_ values in parenthesis are from 16ChRoAG^59^

State	Linear configuration	*T*_0_ (cm^−1^)	Δ*E* (cm^−1^)
1 ^1^A′	X^1^Σ^+^	3418 (3468)	0
2 ^1^A′	1^1^Δ	54 040 (55 491)	70 241
3 ^1^A′	1^1^Π	65 916 (65 815)	75 162
4 ^1^A′	2^1^Π	75 744	82 534
5 ^1^A′	3^1^Π	85 389	86 203

### 2.2 Choice of the basis

Calculations of the photoabsorption cross-section were performed using DVR3D^74^ and Everest.^75^ The original DVR3D program suite, based on the use of the discrete variable representation (DVR) basis, was modified by Zak *et al.*^76^ to calculate rovibronic transitions and tested for SO_2_. The program suite Everest was used to solve the Schrödinger equation using a sinc-DVR basis.^78,79^ The Everest input parameters were tuned to reproduce the energy levels published by Makhnev *et al.*^52^ for the *J* = 0 vibrational energies below 10 000 cm^−1^, the two models agree with an RMSE (root-mean-squares-error) of 0.012 cm^−1^.

**Fig. 3:**
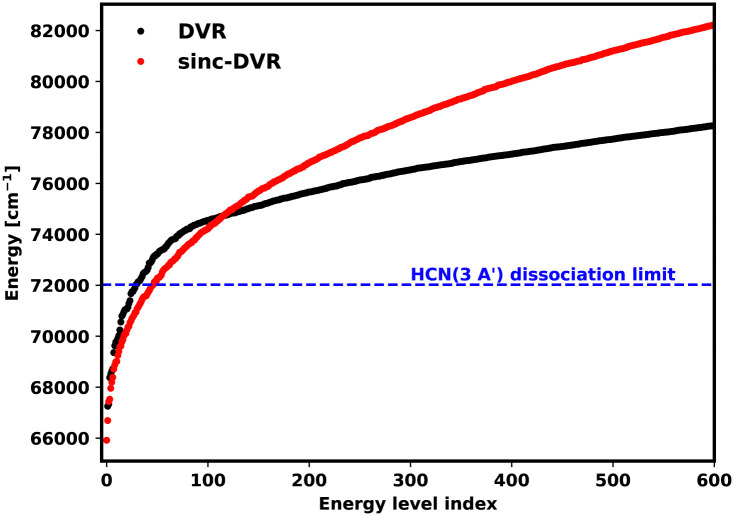
The first 600 vibrational energy levels of HCN (3 ^1^A′). For energies below 70 000 cm^−1^, spherical oscillator DVR and sinc-DVR basis results show a similar trend, but they diverge for higher energies.

For DVR3D, the basis set parameters used for the ground state and excited states must be the same; this is a necessary requirement for calculating the overlap between the two wave functions. DVR3D offers two forms of radial basis functions which can be optimised for a given system: Morse-like oscillators^80^ and spherical oscillators;^81^ standard ground state HCN calculations such as those of Makhnev *et al.*^52^ use Morse-like oscillators for both radial coordinates. Here, to allow for dissociation, we experimented using spherical oscillators for the H–CN coordinate in Jacobi coordinates because they have better space filling properties. However, unlike the sinc-DVR basis which fill a finite, well defined region, the spherical oscillators in principle fill all space but the representation becomes increasingly sparse at large separations. Numerical experiments showed that, while it was possible to recover some of the photodissociation behaviours using spherical oscillators, this was less satisfactory than the results using sinc-DVR basis functions, which have the additional advantage that one can perform repeat calculations with shifted box sizes and obtain useful information.^71,82^ The sinc-DVR basis successfully reproduced the energy levels and transitions observed in HCN, see below. The reason behind the different behaviour of the two basis functions is the way the tridimensional grid is built: the DVR basis set is constructed using a non-uniform, in principle semi-infinite Gaussian quadrature, while the sinc-DVR basis uses a finite and uniform grid. Fig. 3 shows how the distribution of the vibrational levels changes between a spherical oscillator DVR and a sinc-DVR basis for the 3 ^1^A′ dissociative state. The two basis gave different results for energies above 70 000 cm^−1^. The divergence is due to the spherical oscillator DVR basis being unconstrained in the radial coordinates: the basis explores larger regions which means that attempting convergence by increasing the number of points simply results in the production of an increasing, potentially infinite, number of states. For this reason, all calculations in this paper were performed using Everest.

### 2.3 Calculation of the photoabsorption cross-section

In this section, we provide a brief overview of the procedure and steps for generating cross-sections using Everest. A more detailed explanation of the code and underlying algorithms can be found in the original publication by Mitrushchenkov.^75^

The first step required to calculate cross-sections is to generate the vibrational basis set for the electronic states of interest. This is done using the *evvib.e* routine. For each individual state, the corresponding symmetry is specified in terms of the electronic spin (*S*), the molecular term symbol (*A*), and the projection of the orbital angular momentum along the internuclear axis (*Λ*). The atomic compositions, molecular masses and coordinate systems are then specified. For each coordinate, the basis representation, the grid interval and the number of points need to be specified. The bond length coordinates are described using a sinc-DVR basis using the desired number of points (*N*^r^_points_), ranging from *r*_min_ to *r*_max_. The bond angle is described by 
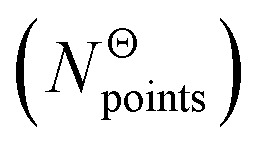
 points using Legendre polynomials ranging from 0 to 180 degrees. This produces the vibrational levels for each *k* (projection of the angular momentum on the *z*-axis) of interest for a Hamiltonian of the desired size.^83^

The second step consists of generating the rotational wave function using *evrot.e*. In this routine, the wave function for the desired *J*_s_ is generated. The rotational embedding is specified in this step.

In the third step, the dipole moment transition matrix is generated with the *evdip.e* routine, using the *x* and *z*-components of the TDMS. The routine *evrli.e* combines the wave function with the transition dipole moments. The *evrs.e* routine generates the molecular spectrum. The output is then transformed and stored in the ExoMol format.^13,84^

A significant advantage of Everest is that potentials, dipole moments, and possible couplings are loaded as dynamic libraries, without the need to recompile the code for each calculation.

**Fig. 4:**
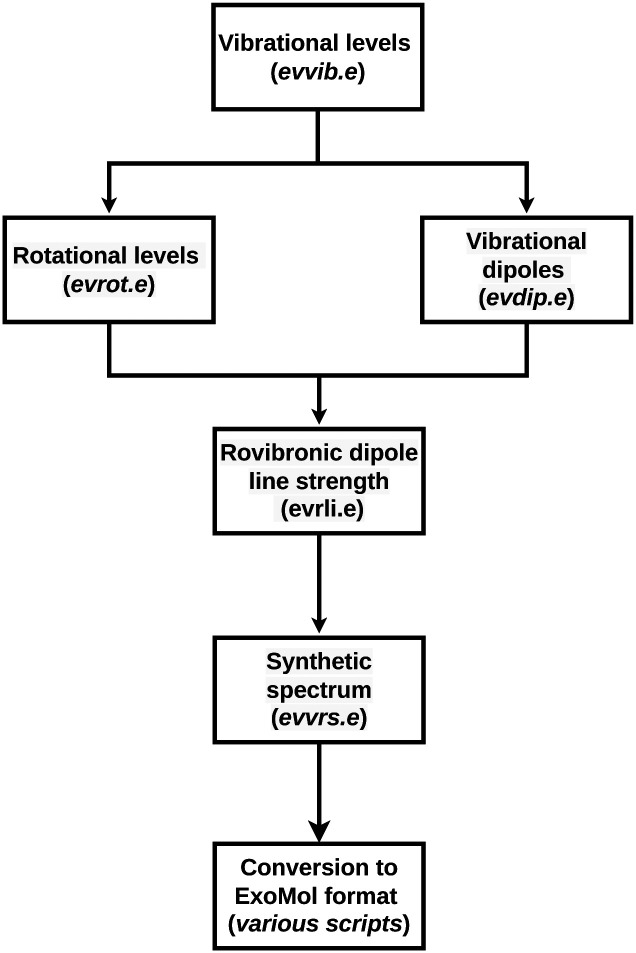
Graphical representation of the workflow used in this paper. For each step, the program used in Everest is written in parenthesis in an italic character.

The Everest parameters used in the present study are reported in Table 2. The first section of the table presents the parameters for the vibrational input. Each state considered in this work is a singlet with A′ symmetry, while *Λ* varies depending on the state considered. Atom names and (atomics) masses are the input with the central atom last. Each calculation was made with a Hamiltonian of size 6000, specifying the first 100 roots for the ground state and the first 1000 roots for the electronically excited states. In the rotational input, *z*-embedding is specified with the *z*-axis lying along the *r*_CN_ bond. For both *r*_CH_ and *r*_CN_, a radial grid spanning from 0.6 Å to 3.5 Å is used to take into account wavefunctions for highly excited vibrational states. At this step, all energy levels below 10^4^ cm^−1^ for the ground state and below 10^5^ cm^−1^ for the excited states are selected. The input runs through the first *N J* and *K* roots. The overall workflow is shown in Fig. 4.

**Table 2:** Input parameters used in the Everest code for HCN

Vibrational input	
*N*_pots_	2
State 1 symmetry	1 A′ *Λ*_1_
State 2 symmetry	1 A′ *Λ*_2_
Atoms	H N C
Masses [Da]	1.007825
	14.003074
	12.000000
Geometry	BLBA
*r*_1_ Grid type	Sinc-DVR
*r*_1_ *N*_points_	50
*r*_1_ Interval [Å]	0.6
	3.5
*r*_2_ Grid type	Sinc-DVR
*r*_2_ *N*_points_	50
*r*_2_ Interval [Å]	0.6
	3.5
*Θ* Grid type	Legendre
*Θ N*_points_	50
*Θ* Interval [degrees]	0
	180
*K*_min_	0
*K*_max_	*N*
Hamiltonian size	6000
Number of roots	100
	1000
Rotational input	
*J* interval	0-*N*
Embedding	*z*-Embedding
Embedding axis	*r*_*x*_
Diagonalisation	Jaco
Hamiltonian size	6000
*E* ^max^_state_ [ cm^−1^]	10 000
	100 000
*K*_min_	0
*k*_max_	*N*
Dipole input	
Number of dipole files	2
Alx	1
Embending	*z*-Embedding
Embending axis	*r*_*x*_

All calculations are performed in the UCL Department of Physics and Astronomy theory cluster using an Intel compiler and an OpenMP parallelisation scheme on 16 nodes.

### 2.4 Cross-section smoothing

With a sinc-DVR basis, one performs a calculation over a finite region (box) defined by internuclear distances *r*_min_, and *r*_max_ which effectively places infinite potential barriers at the boundaries. For diatomic molecules,^14^ it is computationally cheap to repeat the calculation using a sinc-DVR basis generated with an increasing box size. This can form the basis of a stabilization method^85–87^ and is being used by the ExoMol group to study predissociation.^71,73,82^ The choice of the box size sets boundary conditions and controls the density resulting in a discretized continuum spectrum. By adjusting the size of the box by varying *r*_max_, one can sample different areas of the continuum and produce cross-sections with a dependence on the calculation of the box size. For diatomics, it is computationally possible to perform hundreds or thousands of nuclear motion calculations^71^ as individual calculations are relatively quick, requiring, typically, less than an hour of computing time per iteration. This is not the case with triatomic molecules or larger polyatomics, where calculations can take days, weeks, or months per iteration, depending on the size and complexity of the model. As such, performing stabilization-style calculations is currently computationally prohibitive.

A practical alternative to the stabilization method is to compute a discretized continuum spectrum from a single-box calculation and smooth the corresponding temperature-dependent cross-sections, *σ*(*ν*), by applying a uniform Gaussian line profile with an appropriate half-width at half-maximum (HWHM, *α*). This is, at the moment, the only viable method for producing these cross-sections for polyatomics using Everest. The choice of *α* can be constrained by the structure of the discretized spectrum and the shape of the continuum cross-section.

Following Pezzella *et al.*,^14^ the initial cross-sections of 1 ^1^A′→ U electronic transitions are produced with *α* = 10 cm^−1^. Here, U represents any given unbound electronic state. These are the cross-sections which undergo smoothing.

To quantify optimum smoothness, we require that the resulting smoothed cross-section must be continuous and that the minimum value of *α* is used to prevent the over-distribution of intensity to the wings.

To assess the continuity of the cross-sections, one notes that, at their intensity maxima, the cross-sections have isolated turning points. In this region, the intensity distribution is bell shaped and, in general, direct photodissociation to purely repulsive states appears to have this shape as found by Pezzella *et al.*^14,18^

As such, the smoothing of the cross-section can be constrained by the number of expected maxima, *N*_M_. The continuity condition is so defined to be that the resulting, smoothed cross-section should have a number of roots in its first and second differential equal to *N*_M_ and *N*_M_ + 1, respectively, hence, should be twice differentiable.

It is found that there are usually a range of *α* values that meet this condition, and so the minimum value is taken to satisfy the condition on minimum smoothing. Eqn (1) expresses these conditions formally:1
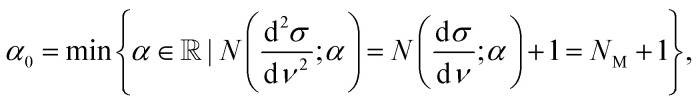
where *N*(*f*) is the number of roots as a function *f*. Due to the discrete nature of this problem, one cannot implement this procedure as a standard minimization problem through treatments like gradient descent and instead it must be solved iteratively.

The numerical method behind this is to establish a range of *α* values over which to test. At each iteration, the first and second forward derivatives are computed as follows:2
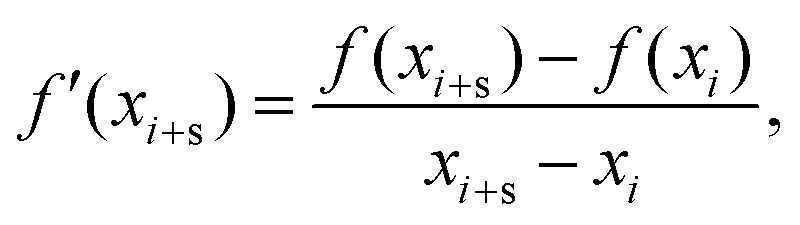
where *s* is the sampling window which is chosen to deal with the numerical noise of producing these differentials. In general, a value of 
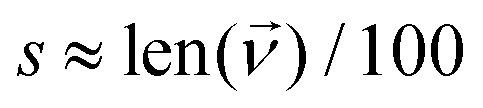
, where 
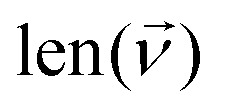
 is the size of the wavenumber grid, is found to be appropriate for both first and second derivatives. Roots are counted by scanning the available smoothed cross-section and finding where the sign of the derivatives change. In areas of low intensity in the initial cross-section, small changes in intensity result in large changes of the gradient. While roots in these regions are not false positives, it is impractical to count them. To counteract this, a threshold value, *K*, is imposed such that a root will only be counted if the smoothed cross-section is *σ̂*(*ν*;*α*) >*K*.

**Fig. 5:**
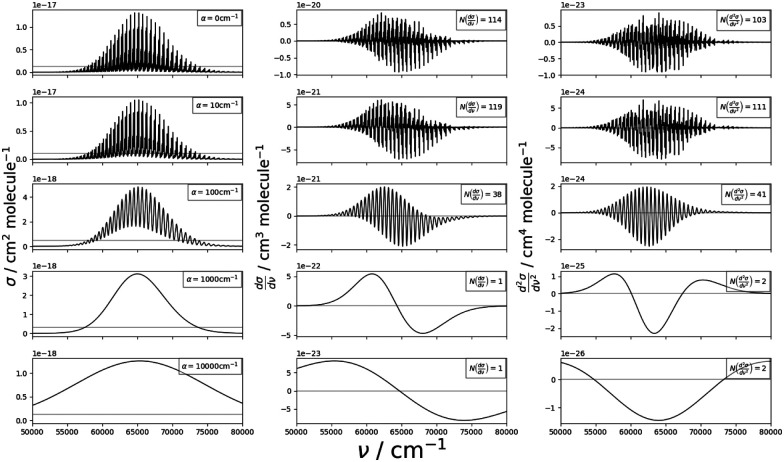
Toy model: Dependence of the shape of a photodissociation cross-section and its first and second derivatives on *α* in the smoothing procedure. Left panels show the smoothed cross-section, middle panels are the first derivatives, and right panels are the second derivatives.

Each iteration checks in the number of counted roots which meet the threshold conditions, upon the continuity condition being met or all test values are used. An example showing how the increasing *α* parameter affects the derivatives and shape of a toy model's cross-section is shown in Fig. 5. As can be seen, both *α* = 1000 cm^−1^ and *α* = 10 000 cm^−1^ meet the continuity condition imposed by eqn (1); however, the shape of the cross-section in the *α* = 10 000 cm^−1^ case does not faithfully represent the shape of the raw cross-section in the top left panel; therefore, one must include the requirement for minimal smoothing. In this dataset, the threshold value was estimated as *K* = max(*σ*)/10.

The benefits of this procedure are that it is simple to implement, requires minimal computational expense, and provides good results in terms of smoothing. A limitation of this method is that upon meeting the aforementioned criteria, smoothing does not tend to be sufficient at higher *ν*. As *ν* increases, the gaps between successive vibrational bands also increases and so a minimal smoothing parameter as defined above would still allow for discrete structures to show through at high *ν*. One could increase *α* to account for this; however, this results in a slight over-smoothing in the lower *ν* region and is more challenging to automate reliably. This is a minor limitation, however, as the temperature dependent effects of these cross-sections will manifest themselves primarily in the lower wavenumber regions.

A Python3 code, ExoSmooth, has been written to automate the procedure of optimizing the HWHM. The program applies Gaussian profiles to the unsmoothed cross-section, starting at a given HWHM and increasing the HWHM by some user-defined amounts at each iteration until the continuity conditions described above are met. This ensures that the minimum amount of smoothing possible is applied to the cross-section. Indeed, as the Gaussian profile is normalised, the integral of the smoothed and unsmoothed cross-sections is equivalent; hence, photodissociation rates calculated with either cross-section are also equivalent. The documented code is made available from https://github.com/exomol. We note that the test performed as part of our initial study on diatomics^14^ showed that photodissociation rates obtained with the smoothed and unsmoothed data were the same, and that comparisons with (rotation-free) full photodissociation calculations also gave essentially the same photodissociation rate at low temperatures.

## 3 Results

### 3.1 Comparison with experiments

Of the four excited states available, there are no experiments available for the 2 A′ ← 1 A′ transition; the 3 A′ ← 1 A′ transition has been explored by different authors,^45,88,89^ thanks to its well defined structure and being isolated from other electronic states; the 4 A′ ← 1 A′ and 5 A′ ← 1 A′ transitions are localised in the same energy region, making difficult to separate the two contributions in the absorption spectrum.^45,89,90^

**Fig. 6:**
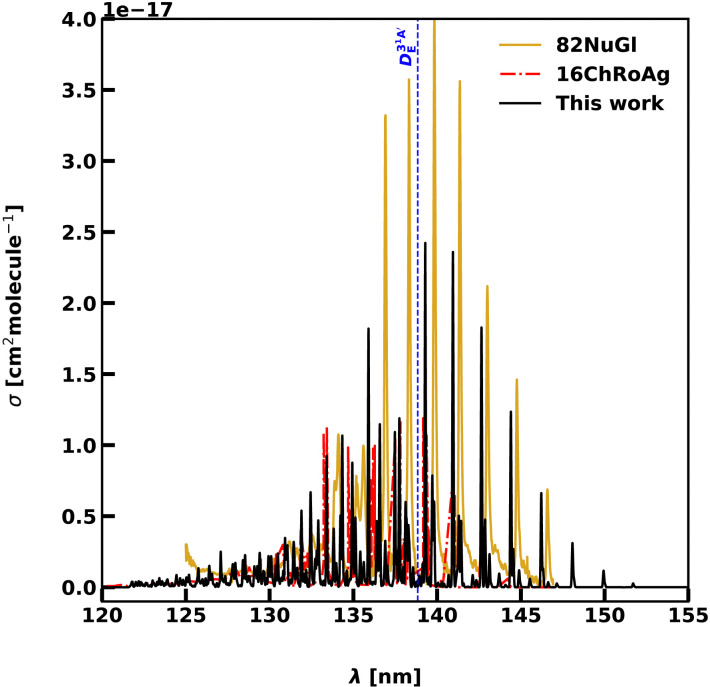
Comparison between our photoabsorption spectrum for the 3 A′ ← 1 A′ transition at *T* = 0 K and *J* = 0,1, with the computed model from 16ChRoAg^59^ and the experiments from 82NuGl.^89^ The blue dashed line separates the bound–bound spectrum at longer wavelengths from the photodissociation region at shorter wavelengths.

The 3 A′ is the only excited state which shows a clear vibrational progression. These levels make it possible to evaluate the quality of the potential used and the overall validity of the model. The quality of our calculations is tested against the computational results of 16ChRoAg and the experiments of Nuth *et al.* (82NuGl).^89^ The first two models are computed including only the *J* = 1 ← 0 transitions, which effectively models *T* = 0 K. The results are shown in Fig. 6. The peak positions agree, in terms of relative intensities between the bending progression, with the measurements of 82NuGl, while the calculations of 16ChRoAg tend to be blue shifted with respect to the other sources. The cross-section magnitudes agree with 16ChRoAg up to *v*_2_ = 6, while, for *v*_2_ = 7,8 we observe a major splitting between the *K* components compared to the other two methods, leading to a lower cross-section peaks than 16ChRoAg. The splitting becomes evident for *v*_2_ ≥ 9 in all sources. A clear drop of the cross-section is observed for energies higher than the 3 A′ dissociation barrier at 138.84 nm (72 025 cm^−1^). The absorption spectrum presents both bound–bound features, due to the bending progression, together with an increasing dissociative character raising from dissociative threshold at wavelengths shorter than 138.84 nm.

Table 3 reports measured vibrational band centers for the 3 A′(0,*v*_2_,0) ← 1 A′(0,0,0) bands from MacPherson & Simons (78MaSi),^88^ Lee (80Lee),^45^ 82NuGl and our values at *T* = 0 K. The experimental vibrational values vary by 10 to 70 cm^−1^ between the different measurements. In our calculations, the ground state transition is shifted by 46 cm^−1^ from 80Lee with a RMSE of 68 cm^−1^. The RMSE is 64 cm^−1^ compared to that of 78Masi, while a lower RMSE of 47 cm^−1^ is found when comparing the measurements of 82NuGl.

**Table 3:** Position for the peaks of the HCN (3^1^A′ (0, *v*_2_, 0) ← *X̃*′Σ^+^ (0,0,0)). Experimental values are from 78MaSi,^88^ 80Lee^45^ and 82NuGl.^89^ Transition frequencies and the RMSE of the observed calculated results are in cm^−1^

*v*_2_	*ν*_78MaSi_	*ν*_80Lee_	*ν*_82NuGl_	*ν*_This work_
0	—	65 647	—	65 693
1	—	66 520	—	66 691
2	—	67 376	—	67 434
3	—	68 227	68 217	68 194
4	69 085	69 085	69 080	68 997
5	69 950	69 940	69 935	69 864
6	70 750	70 746	70 746	70 701
7	71 515	71 515	71 515	71 516
8	72 280	72 290	72 296	72 276
9	73 035	73 041	73 046	73 047
10	73 770	73 746	73 741	73 738
11	74 570	74 555	74 566	74 600
12	75 370	75 432	75 438	75 501
13	—	76 214	76 220	76 294
14	—	—	76 938	76 966
RMSE	64	68	47	

**Fig. 7:**
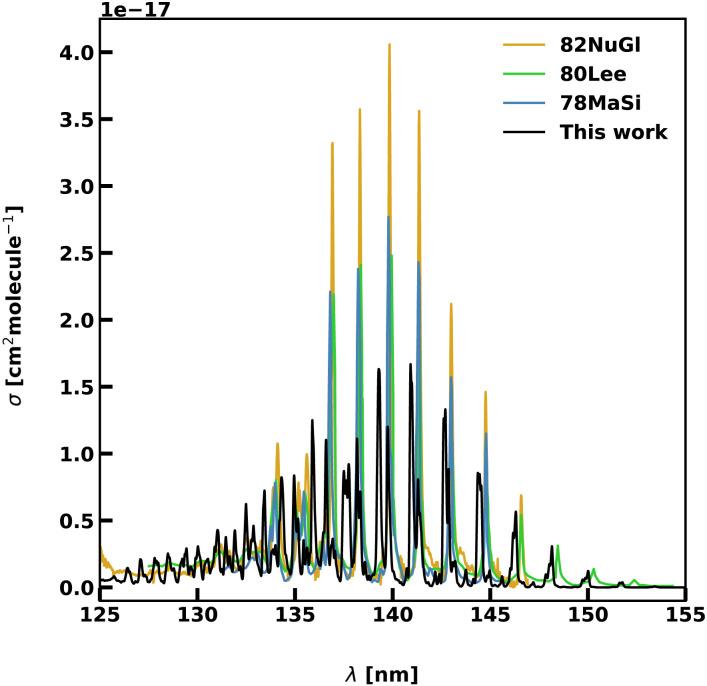
Photoabsorption spectra of the 3 A′ ← 1 A′ transition. Our computed spectrum (0 < *J* < 25) is in black, experimental spectra are in blue for 78MaSi,^88^ green for 80Lee,^45^ and gold for 82NuGl.^89^ The photodissociation region is found at wavelengths shorter than 138.84 nm.

Fig. 7 compares a computed spectrum of the 140 nm band, calculated at 300 K with 0 < *J* ≤ 50, with the experiments. A partition function of 899.7 calculated using this rotational interval is in agreement within 99% with the values reported in the ExoMol database as taken from the Harris line list for HCN^91^ at this temperature, suggesting a near complete solution. The ExoMol partition function is based on rovibrational energies with *J*_max_ = 60. The magnitudes of the cross-sections are lower than those measured by 78MaSi and 80Lee suggesting that the 16ChRoAg transition dipoles are too small, while the cross-sections from 82NuGl are consistently higher than those in all experiments. For higher vibrational levels, we observe that the computed spectra show a higher level of rotational splitting than experiments. The vibrational progression of *v*_2_ = 8 at 137.74 nm is spread along a 1 nm interval with a large splitting between the rotational components.

**Fig. 8:**
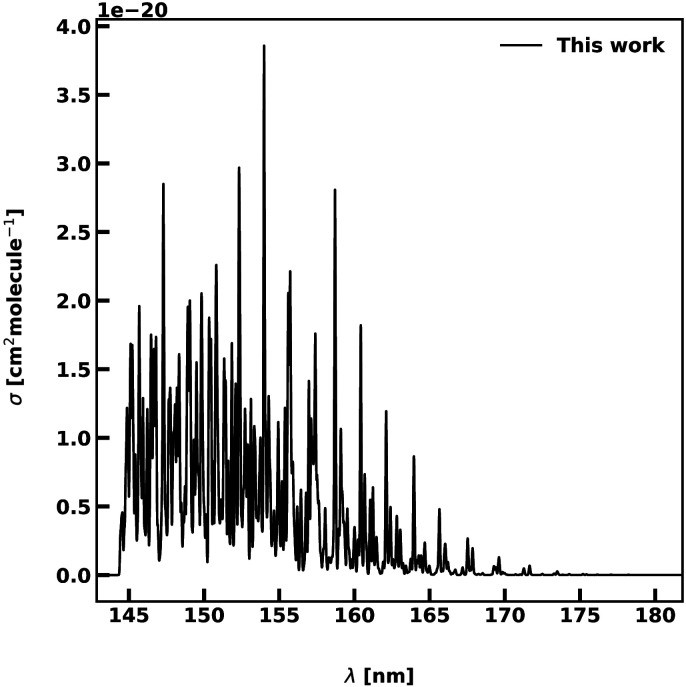
Photoabsorption spectra of the 2A′ ← 1 A′ transition. The spectrum is computed at *T* = 0 K. The photodissociation regime is found for wavelengths below 155 nm.

The 2 A′ state corresponds to one component of the ^1^Δ state at linear configurations, making transitions to this state forbidden at linear geometries and weak for bent structures. The intensities are of the order of 10^−20^ cm^−1^, being between 2 and 3 order of magnitudes lower than the other transitions. Fig. 8 shows our computed spectrum between 145 and 175 nm when using 1000 roots. A small vibrational progression is observed for wavelengths longer than 155 nm, followed by a bound-free region below this limit.

**Fig. 9:**
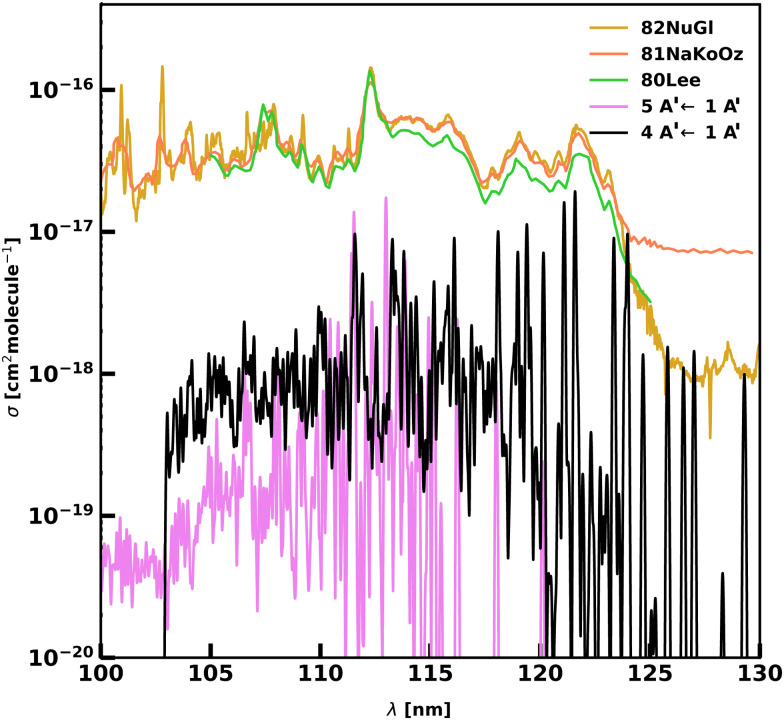
Bound–bound photoabsorption spectra of the 4 A′ ← 1 A′ (black) and 5 A′ ← 1 A′ (pink) transitions *versus* the experiments using a logarithmic *y*-scale. Experimental spectra are in green for 80Lee, orange for 81 NaKoOz and gold for 82NuGl. The computed spectra are consistently lower than that measured by the experiments.

Table 4 and Fig. 9 show our results for the 4 A′ ← 1 A′ and the 5 A′ ← 1 A′ bands compared to the experiments from 81NaKoOz and 82NuGl. Our peak assignments are made by performing calculations at *T* = 0 K. The experiments, however, report only the vibrational levels without information on the rotational levels. A small shift between the energy levels can be expected, as at room temperature the maximum of the cross-section will be found for higher rotational states. 81NaKoOz identifies 6 vibrational states within the two electronic state, and we found that our levels are consistently higher than theirs with an RMSE of 35.1 cm^−1^. We get better agreement with the 17 levels measured by 82NuGl, with a RMSE of 14.0 cm^−1^. A larger disagreement is found for the *v*_2_ = 2 state, with a difference of 157 cm^−1^. Excluding this state, the RMSE decreases to 10.0 cm^−1^. However, we get poor agreement with the magnitudes of the cross-sections; our computed magnitudes are lower by a factor of 5–10 in the 100–130 nm region. It is likely that the computed TDMSs are too small.

**Table 4:** Vibrational band centers 4 A′ ← 1 A′ and 5 A′ ← 1 A′ bands: Comparison between theory (this work) and experiment, from 82NuGl^89^ and 81NaKoOz^90^

*v*_1_ *v*_2_ *v*_3_	81NaKoOz	82NuGl	This work
4 A′ ← 1 A′			
000	82 100	82 217	82 243
020	—	83 222	83 227
001	83 960	83 991	84 041
002	—	85 948	86 000
5 A′ ← 1 A′			
000	88 980	89 063	89 013
010	—	89 718	89 666
020	90 170	90 400	90 243
001	90 930	90 992	91 025
011	—	91 659	91 706
100	92 070	92 039	92 113
021	—	92 396	92 508
110	—	92 653	92 682
002	—	92 963	92 936
031	—	93 101	93 090
120	—	93 396	93 384
130	—	94 171	94 159
200	—	95 057	95 057

### 3.2 Integrated intensities

The calculation of the integrated intensity (*I*) is a second method to assess the quality of our calculations. The method itself is the integration of the photodissociation cross-sections over a specific wavelength range (*λ*_1_ and *λ*_2_):3
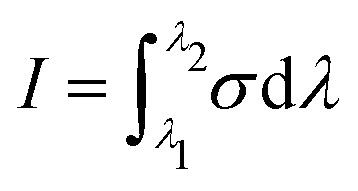


The MPI-Mainz UV/VIS Spectral Atlas (ref. 92)‡‡https://www.uv-vis-spectral-atlas-mainz.org/uvvis contains 4 different measured electronic spectra of HCN. The intensities of the 3 A′ ← 1 A′ are integrated from the measurement from 78MaSi^88^ and 80Lee.^45^ Upper state excitations are measured from 80Lee, 81NaKoOz^90^ and 82NuGl.^89^ The Mainz database simply reports the overall spectrum over the experimental wavelength range, making it impossible to make a one to one comparison with our results in terms of individual transitions.

The integrated intensities for the 3 A′ ← 1 A′ bands from our model (1.57 × 10^−14^ cm molecule^−1^) and the spectrum extracted from 16ChRoAg^59^ (1.40 × 10^−14^ cm molecule^−1^) are within 12%, showing overall agreement between the two computational models. For these calculations, only the *J* = 1 ← 0 transitions are considered. At room temperature (300 K), the calculated band intensity, with *J* varying from 0 to 25, is 3.01 × 10^−14^ cm molecule^−1^. Experiments from 78MaSi, 80Lee, and 82NuGl show higher intensities than the computed ones, by a factor of 1.5 or 6, depending on the source. Values are reported in Table 5.

**Table 5:** Integrated intensities from experiments and calculations for HCN electronic transitions. Lee^45^ and MacPherson & Simons^88^

Source	*T* (K)	*J*	*I* (cm molecule^−1^)
**2** A′ ← **1** A′			
**Computed**			
This work	0	0,1	5.56 × 10^−17^
This work	300	0,20	8.36 × 10^−18^
16ChRoAg^59^	—	0,1	4.17 × 10^−17^
**3** A′ ← **1** A′			
**Computed**			
This work	0	0,1	1.57 × 10^−14^
This work	300	0,25	3.01 × 10^−14^
16ChRoAg^59^	—	0,1	1.40 × 10^−14^
**Experiments**			
80Lee^45^	Room temperature	—	8.33 × 10^−14^
82NuGl^89^	Room temperature	—	1.86 × 10^−13^
78MaSixx^88^	Room temperature	—	4.41 × 10^−14^
**4** A′ ← **1** A′			
**Computed**			
This work	0	0,1	2.18 × 10^−14^
This work	300	0,20	2.67 × 10^−14^
**5** A′ ← **1** A′			
**Computed**			
This work	0	0,1	6.07 × 10^−15^
This work	300	0,20	8.71 × 10^−15^
**Upper states**			
**Experiments**			
80Lee^45^	Room temperature	—	2.50 × 10^−12^
82NuGl^89^	Room temperature	—	1.08 × 10^−12^
81NaKoOz^90^	Room temperature	—	8.20 × 10^−13^

The integrated intensity of the 2 A′ ← 1 A′ band for our model agrees with the value from 16ChRoAg. The intensity decreases with the increase of temperature and rotational states, from 5.56 × 10^−17^ cm molecules at 0 K to 8.36 × 10^−18^ at 300 K.

There is no experimental measurement for individual higher states; rather, they are measured collectively. The intensities are of the order of magnitude of 10^−12^ cm molecule^−1^. These values are higher than our computed values by a factor between 30 and 90, depending on the experimental source. The cause of the underestimation of the cross-sections and intensities with respect to the experiment was ascribed by 16ChRoAg^59^ to excited states possibly stealing intensity from dark states, not considered in their work or ours, through coupling to other states. It would seem more likely that these discrepancies arise from the computed PESs and TDMs: the electronic intensities are very sensitive to the position and shape of the upper state; coupled with a possible underestimation of the transition dipole moments.

The causes of the underestimation of the cross-sections and intensities with respect to the experiment was ascribed by 16ChRoAg for two possible reasons:^59^ these excited states might steal intensity from dark states, not considered in this work, through coupling from other states. Other possible reasons of these discrepancies are in the computed PESs and TDMs: the electronic intensities are very sensitive to the position and shape of the upper state; coupled with a possible underestimation of the transition dipole moments.

### 3.3 Temperature dependence of the 3 A′ ← 1 A′ band

**Fig. 10:**
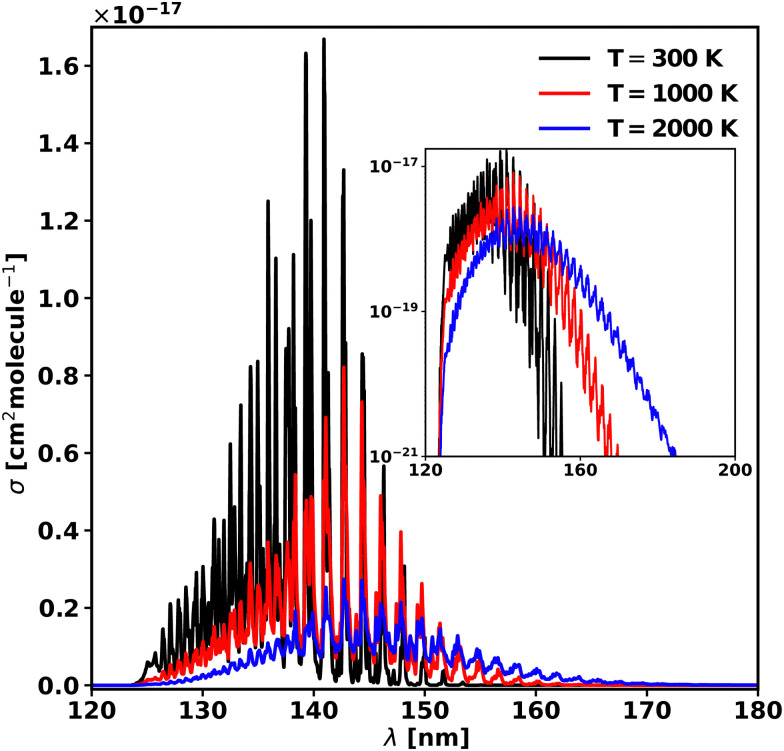
Photoabsorption spectra of the 3 A′ ← 1 A′ transition at 300, 1000, and 2000 K. The main plot is in a linear scale, while the inset is the same plot but in the logarithmic scale.

As in the case of diatomic molecules, the increase in temperature leads to a change in the shape of the cross-sections. In order to understand this effect, the cross-sections were computed at 300, 1000 and 2000 K and are plotted in Fig. 10 in linear and logarithmic forms. The calculated partition function of 50 681.8 at 2000 K is 99% of the value in the Harris line list for HCN^91^ from the ExoMol database. The increase of temperature corresponds to an increase of the background continuum, the well separated vibrational progression up to 2000 K. The maximum of the cross-section, corresponding to *v*_2_ = 7 peak, is red shifted by 1.79 nm (890 cm^−1^) from 300 K to 1000 K, and it remains constant up to 2000 K. The cross-section height decreases by a factor of 6 when the temperature reaches 2000 K from 300 K. These values are collected in Table 6. However, importantly for modelling applications, the threshold to photodissociation cross-section drops significantly with temperature, as observed previously in our study of diatomics,^18^ which can lead to very large increases in the effective rate of dissociation.

**Table 6:** Temperature dependence of various spectroscopic properties (*σ*_max_, *λ*_max_) of the 3 A′ ← 1 A′ transition

*T* (K)	*σ*_max_ (cm^2^ molecule^−1^)	*λ*_max_ (nm)
300	1.67 × 10^−17^	140.92
1000	8.22 × 10^−18^	142.71
2000	3.04 × 10^−18^	142.71

## 4 Conclusions

In this work, we explore the possibility of studying the direct photodissociation *via* dissociative potential surfaces and indirect photodissociation *via* quasi-bound excited states of triatomic molecules. The distinction between these two mechanisms is less clear cut in polyatomics than in diatomics. Our approach is based on solving the time-independent Schrödinger equation using variational nuclear motion calculations provided by adapting existing programs. We chose HCN as a test molecule, as it is a well-studied molecule experimentally and various computational studies. We performed the calculations using Everest, a program suite developed from DVR3D with some advantages when studying unbound states. This is because the latter code utilises a sinc-DVR basis, which provides better coverage of the dissociating coordinates compared to spherical oscillators, which tend to become increasingly sparse at large interatomic separations.

Our method is able to reproduce the experiments and the original work from 16ChRoAg.^59^ The discrepancies between experiments and our calculations can be explained by our use of an adiabatic description of the PESs and issues with the calculated transition dipole moments. Our cross-sections and intensities, like as in the previous study by 16ChRoAg, are underestimated with respect to the experiments. In their paper, 16ChRoAg mention as possible reasons for these discrepancies the lack of couplings in the model and the difficulty of computing accurate transition dipole moments involving electronically excited states.

The temperature dependence of the cross-section has been tested on the 3 A′ ← 1 A′ transitions at three different temperatures. A shift in the cross-section peak and a decrease of the cross-section height plus a lower of the photodissociation threshold are observed with increasing temperatures, as found for diatomic molecules.^14,18^

The next steps will be to produce a complete photodissociation model for HCN, performing electronic structure calculations for the states not included in this work and refining the available potential energy and transition dipole surfaces to recover the low-temperature experimental results. This model will then be used to provide cross-sections over an extended range of molecular temperatures. We plan to use our newly developed method to study other important molecules such as H_2_O and H_2_S. Non-adiabatic couplings between potential surfaces can provide an important mechanism for photodissociation. We note that Everest allows the implementation of different forms of surface couplings, including non-adiabatic effects. These features have been successfully tested on bound–bound electronic transitions of CaOH, which are characterised by both Renner–Teller and spin–orbit coupling effects, as demonstrated by Owens *et al*.^77^ This is a supplementary reason for our choice of Everest. Calculations on the photodissociation of H_2_S which include the explicit treatment of non-adiabatic couplings using Everest are currently in progress.

## Data availability

The data for this article are contained in the article: numerical values for the figures are available from the authors. The documented codes, ExoSmooth and DVR3D, are available from https://github.com/exomol.

## Conflicts of interest

There are no conflicts to declare.
